# iAssembler: a package for *de novo *assembly of Roche-454/Sanger transcriptome sequences

**DOI:** 10.1186/1471-2105-12-453

**Published:** 2011-11-23

**Authors:** Yi Zheng, Liangjun Zhao, Junping Gao, Zhangjun Fei

**Affiliations:** 1 Department of Ornamental Horticulture, China Agricultural University, Beijing 100094, China; 2 Boyce Thompson Institute, Cornell University, Ithaca, NY 14853, USA; 3 USDA Robert W. Holley Center for Agriculture and Health, Tower Road, Ithaca, NY 14853, USA

## Abstract

**Background:**

Expressed Sequence Tags (ESTs) have played significant roles in gene discovery and gene functional analysis, especially for non-model organisms. For organisms with no full genome sequences available, ESTs are normally assembled into longer consensus sequences for further downstream analysis. However current *de novo *EST assembly programs often generate large number of assembly errors that will negatively affect the downstream analysis. In order to generate more accurate consensus sequences from ESTs, tools are needed to reduce or eliminate errors from *de novo *assemblies.

**Results:**

We present iAssembler, a pipeline that can assemble large-scale ESTs into consensus sequences with significantly higher accuracy than current existing assemblers. iAssembler employs MIRA and CAP3 assemblers to generate initial assemblies, followed by identifying and correcting two common types of transcriptome assembly errors: 1) ESTs from different transcripts (mainly alternatively spliced transcripts or paralogs) are incorrectly assembled into same contigs; and 2) ESTs from same transcripts fail to be assembled together. iAssembler can be used to assemble ESTs generated using the traditional Sanger method and/or the Roche-454 massive parallel pyrosequencing technology.

**Conclusion:**

We compared performances of iAssembler and several other *de novo *EST assembly programs using both Roche-454 and Sanger EST datasets. It demonstrated that iAssembler generated significantly more accurate consensus sequences than other assembly programs.

## Background

Expressed sequence tags (ESTs) are short sub-sequences of transcribed genes and have been extensively used for gene discovery [1] and digital expression analysis [2]. Recent advances in next-generation sequencing (NGS) technologies allow sequencing of large-scale ESTs in an efficient and cost-effective way. One of these technologies, Roche-454 massive parallel pyrosequencing platform [3], has been widely used to sequence transcriptomes of various non-model organisms [4-9] due to its relatively long reads generated (currently ~400 bp) that greatly facilitates *de novo *assembly.

Several *de novo *assembly programs such as CAP3 [10], MIRA [11], TGLCL [12], Phrap [13], and Newbler (Roche) have been developed to assemble EST sequence reads into longer contigs. However, most of these programs are primarily developed for genome sequence assembly, even their transcriptome assembly modes have not been fully optimized and two types of assembly errors are frequently observed: 1) type I error-ESTs derived from alternatively spliced transcripts or paralogs are incorrectly assembled into one transcript; 2) type II error-ESTs derived from the same transcript fail to be assembled together. We have investigated these two types of errors in the Dana-Farber Cancer Institute (DFCI) Plant Gene Index [14], which was created by assembling Sanger ESTs into unigenes using TGICL [12], as well as several other EST databases. Surprisingly, we found that a large number of unigenes with significant overlap (e.g., > 500 bp) and high sequence identity (e.g., > 99%) were not assembled together, such as TC219875 and TC221582 in the DFCI Tomato Gene Index (Additional file 1), and ESTs with significant sequence differences were assembled together, e.g., AW218649 and TC237370 (< 92% identity; Additional file 1), and AW031810 and TC223103 (alternative splicing; Additional file 1) in the DFCI Tomato Gene Index. The assembly error rates are also high for Roche-454 ESTs as we have constantly observed that large portion of Roche-454 unigenes contain assembly errors after reanalyzing several published datasets. Recently Kumar and Blaxter [15] recommended an assembly strategy that involves combining differently optimal assemblies from multiple programs. This strategy can generate better assemblies by taking advantage of advantages of different assembly programs; however it still contains significant number of mis-assemblies. To date, no program is available that can efficiently identify and correct the two types of errors described above.

In this paper we describe iAssembler, a package that can efficiently assemble large-scale EST datasets and automatically identify and correct assembly errors. We demonstrate the utility and performance of this program by performing assemblies on different EST datasets with different sets of parameters.

### Implementation

iAssembler is implemented in Perl and can be executed under either 32-bit or 64-bit Linux systems with Bioperl [16] installed. Although MIRA, CAP3 and NCBI megablast [17] programs are required by iAssembler, they are already integrated into the iAssembler package for user's convenience. Thus iAssembler is easy to install and simple to use.

### Architecture of iAssembler

**Figure 1:**
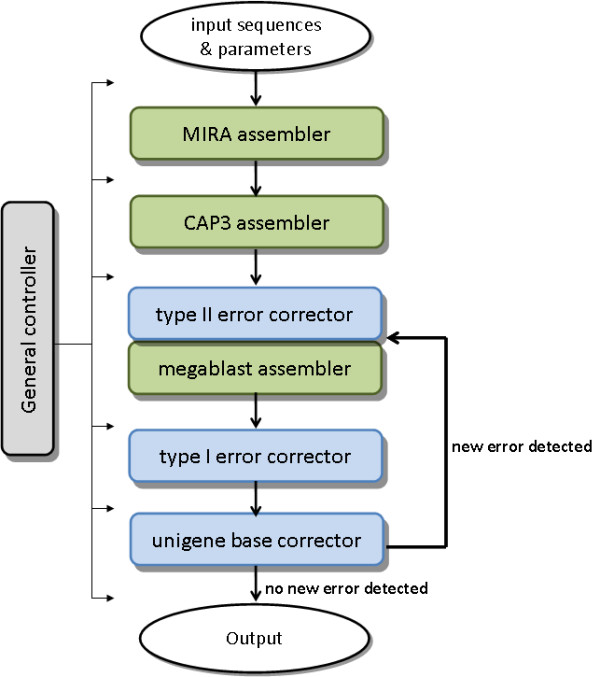
**Architecture and workflow of iAssembler**.

iAssembler employs an iterative assembly strategy and automated assembly error corrections to deliver highly accurate *de novo *assemblies of EST sequences. As shown in Figure 1, iAssembler contains seven major functional modules: general controller, MIRA assembler, CAP3 assembler, megablast assembler, type I error corrector, type II error corrector, and unigene base corrector. These seven modules can be grouped into three categories: controller (general controller), assembler (MIRA assembler, CAP3 assembler, and megablast assembler), and error corrector (type I error corrector, type II error corrector, and unigene base corrector). The general controller module controls the overall running process of iAssembler. It ensures correct parameters, which are defined by users, are passed to each assembler and error corrector, and processes output files from preceding modules to ensure the file formats are compatible with current modules. It also controls iterations of assemblies and error corrections and ensures iterations stop if no new assembly errors are detected (Figure 1). MIRA and CAP3 assemblers are Perl scripts wrapping standalone programs MIRA and CAP3, respectively. They are used to generate initial assemblies of EST sequences. Megablast assembler uses alignment information of two sequences generated by megablast program to assemble them into one contig. This assembler is used to assemble sequences from same transcripts that fail to be assembled by either MIRA or CAP3. Error correctors in iAssembler include type I and II assembly error correctors and the unigene base corrector. They contain functions to identify and correct all possible assembly errors left by MIRA and CAP3 (see below for details). It is worth noting that the megablast assembler and type II error corrector are integrated modules as the identified type II errors are immediately corrected by the megablast assembler.

### Error corrections in iAssembler

The unique feature of iAssembler is its ability to detect and automatically correct all possible assembly errors. Following initial assemblies by MIRA and CAP3, all-versus-all pairwise sequence alignments of resulting unigenes are performed using the NCBI megablast program. Unigenes whose overlapped sequence length and identity, and overhang length meet user-defined cutoffs are identified as type II assembly errors, i.e., sequences from same transcripts fail to be assembled together. The megablast assembler then utilizes the pairwise sequence alignment information to join the unigenes into new contigs. Next, the type I error corrector module maps individual EST members to their corresponding contigs using megablast. ESTs that have sequence similarities to their corresponding contigs less than and/or overhang lengths larger than the corresponding user-defined cutoffs are identified as type I assembly errors, i.e., two different transcripts are incorrectly assembled together. These misassembled ESTs are then extracted by the type I error corrector and together with unigenes derived from the current round of assembly and error correction, are used as the input sequences in the next round of assembly and error correction (Figure 1).

The iterative assembly strategy employed by iAssembler can result in loss of accuracy in final unigene base calling since later assemblies are performed on unigenes generated from previous assemblies, instead of ESTs; thus during assemblies by CAP3 and megablast assemblers, the information of depth of coverage by individual EST members at each unigene position will be lost and thus not used in base calling of assembled sequences. This will cause significant number of wrongly called bases in unigenes. iAssembler provides a unigene base error correction module (Figure 1) which reassigns each individual base sequence of unigenes according to the SAM [18] output file (generated by iAssembler) which contains detailed alignment information of individual ESTs to their corresponding unigenes. The most frequent base covering a specific position will be assigned to that position of the unigene.

Following corrections of type I and II assembly and unigene base calling errors, iAssembler reevaluates the resulting unigenes and identifies and corrects new assembly and base calling errors. The error identification and correction steps will be iterated until no new errors can be identified or corrected.

**Figure 2:**
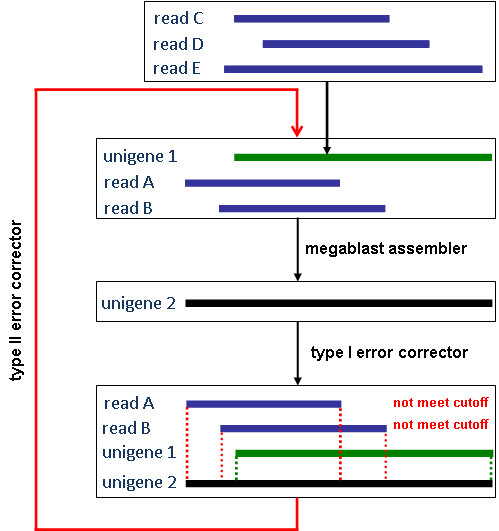
**Example of assembly errors that can't be correct by iAssembler**.

It is worth noting that not all identified assembly errors can be corrected by iAssembler. A simple such example is illustrated in Figure 2. Suppose that unigene 1 is assembled from reads C, D and E and sequence identities between reads A, B and unigene 1 meet the user-defined cutoff, then during type II error correction step, these three sequences (read A, B and unigene 1) can be assemble into one unigene, 2, by the megablast assembler module and each base of unigene 2 is called based on the most frequent base covered by the five reads, A, B, C, D and E. Following assembly, iAssembler will perform type I assembly error detection using its type I error corrector module by aligning reads A, B and unigene 1 to unigene 2, respectively. Now it is possible that sequence identities between read A and unigene 2, and read B and unigene 2 both fail to meet the cutoff. iAssembler will then treat reads A and B, as well as unigene 1, as unigenes to perform type II error correction in the next round of iteration. As shown in Figure 2, this will generate an endless loop and the error will never be corrected. iAssembler will stop the loop if the identified errors are not new ones.

## Results

iAssembler is designed to generate highly accurate assemblies of EST sequences by performing iterative assembly strategy and automated error detection and correction. The three assemblers in iAssembler, MIRA, CAP3 and megablast assemblers, are all base on the overlap-layout-consensus strategy thus iAssembler is applicable for ESTs with relative long sequences, such as those generated using Sanger and/or Roche-454 platforms.

### Workflow of iAssembler

The workflow of iAssembler is shown in Figure 1. iAssembler takes Roche-454 and/or Sanger EST sequences in FASTA format as its input. Before being fed to iAssembler, the EST sequences need to be cleaned by removing low quality regions and known sequence contaminations (e.g., adapters, vectors, and rRNAs) to avoid misassemblies and misinterpretations. This can be achieved by using sequence cleaning programs such as SeqClean [19] or LUCY [20]. It is worth noting that iAssembler itself does not contain functions to clean and trim raw EST sequences.

Cleaned EST sequences are first supplied to iAssembler with appropriate user-defined parameters. iAssembler first employs MIRA to assemble EST sequences, followed by assembling the resulting MIRA unigenes using CAP3. These two open source assemblers were chosen because we have observed that MIRA is efficient in handling large-scale and relatively short Roche-454 reads while CAP3 can complement MIRA by correcting certain type II assembly errors. Following initial assemblies by MIRA and CAP3, type II assembly errors (unigenes belonging to same transcripts) are then identified by performing all-versus-all pairwise sequence alignments of the resulting unigenes using the NCBI megablast program. iAssembler then utilizes the pair-wise alignment information to assemble these unigenes into new contigs using the megablast assembler module. Next, iAssembler identifies type I assembly errors by aligning individual EST members to their corresponding unigenes. The misassembled ESTs, whose alignments to their corresponding unigenes do not satisfy cutoffs of user-specified parameters such as minimum percent identity or maximum overhang, were then extracted and used in the next round of assembly and error correction. Finally, unigene base calling errors are corrected based on alignment information of individual ESTs to their corresponding unigenes contained in the SAM output file. iAssembler iterates through error identification and correction steps until no new errors can be identified or corrected.

The main output of iAssembler includes 1) the final assembled unigene sequence file in FASTA format, 2) a text file summarizing the statistics of alignments of ESTs against their corresponding unigenes, which provides necessary information to assess the quality of the assembly, and 3) a file containing detailed alignment information of individual EST sequences against their corresponding unigenes in SAM format. SAM format is a generic alignment format for storing read alignments against reference sequences [18] and has been adopted by most next-generation sequence alignment and assembly programs. SAM files can be processed and manipulated by SAMtools, for example, SAMtools can convert SAM files into BAM files, the binary form of SAM files, for significant fast accessing and hard disk saving, and can generate pileup output from SAM files for SNP detection [18]. SAM files can also be viewed by several next-generation sequence assembly visualization programs including IGV [21] and Tablet [22].

### Evaluation of iAssembler

We compared the performance of iAssembler to that of several commonly used EST assembly programs including MIRA, CAP3, TGICL, Phrap, and Newbler. An olive EST dataset generated using the Roche-454 platform as described in Alagna et al. [23] and a tomato Sanger EST dataset collected from NCBI dbEST database [24] were first used for the evaluation. Both EST datasets were cleaned by removing adaptor, vector, and *E. coli *genome sequences, which resulted in a total of 246,993 olive ESTs with an average length of 196 bp and 362,445 tomato ESTs with an average length of 579 bp. EST assemblies were performed using a single CPU on a server with six Quad-core 2.93 GHz Intel Xeon processor and 64 GB of RAM. The following parameters were used for all the tested assembly programs, if applicable: minimum overlap length of 40 bp, minimum overlap percent identity of 97%, and maximum overhang length of 30 bp. Detailed commands and parameters used for these assemblers are listed in Table 1.

**Table 1:** Command and parameters used for evaluating EST assembly programs

Program	Command and parameters
iAssembler	iAssembler.pl -i input_est -h 40 -e 30 -p 97 -d -o output ("-e 10" for Arabidopsis)
CAP3	cap3 input_est -o 40 -y 30 -p 97 -f 6 -s 251 ("-y 10" for Arabidopsis)
TGICL	tgicl input_est -l 40 -v 30 -p 97 ("-v 10" for Arabidopsis)
MIRA (olive)	mira -project = project -fasta = input_est -job = denovo, est, normal,454 -notraceinfo -GE:not = 1 454_SETTINGS -LR:wqf = no -AS:epoq = no:mrl = 30 COMMON_SETTINGS -AS:nop = 4 -SK:not = 1:pr = 97 -CL:pec = no 454_SETTINGS -AL:mo = 40:mrs = 97
MIRA (tomato and Arabidopsis)	mira -project = project -fasta = input_est -job = denovo, est, normal, sanger -notraceinfo -GE:not = 1 SANGER_SETTINGS -LR:wqf = no -AS:epoq = no:mrl = 30 COMMON_SETTINGS -AS:nop = 4 -SK:not = 1:pr = 97 -CL:pec = no SANGER_SETTINGS -AL:mo = 40:mrs = 97
Phrap	phrap input_est -ace
Newbler	runAssembly -cdna -urt -notrim -ml 40 -mi 97 -o output input_est

As shown in Table 2 and 3, the final assemblies of both tomato and olive ESTs using MIRA, CAP3, TGICL, Phrap and Newbler contained large amount of type II errors. This indicated that significant redundancies existed in these assemblies. iAssembler was able to correct the majority of these errors, with very few errors left. As a result, iAssembler produced fewer and significant longer unigenes than other assemblers except Phrap and Newbler. The longer unigene length of Phrap and Newbler assemblies is due to the significant more type I assembly errors they generated, which incorrectly assembled different transcripts into one longer gene (Table 2 and 3). MIRA, CAP3 and TGICL assemblies also contained significant number of type I assembly errors, especially MIRA when run under Sanger settings (Table 2), while iAssembler only left several type I errors.

**Table 2:** Performances of assembly programs with tomato Sanger ESTs (minimum overlap: 40 bp, minimum overlap percent identity: 97%, maximum overhang: 30 bp)

		iAssembler	CAP3	MIRA	TGICL	Phrap	Newbler
No. unigenes		53,734	89,590	84,993	51,502	43,434	49,792
Average unigene length (bp)		920.6	735.2	741.4	920.1	963.7	997.7
No. type I errors	identity < 97%	5	85	26,224	2,602	11,223	8,059
	overhang > 30 bp	3	156	8,282	5,743	34,148	21,540
No. type II errors		254	14,396	12,075	3,036	3,909	5,868
Total assembly errors		262	14,637	46,581	11,381	49,280	35,467
Run Time (minute)		634	369	230	450	175	42

**Table 3:** Performances of assembly programs with olive Roche-454 ESTs (minimum overlap: 40 bp, minimum overlap percent identity: 97%, maximum overhang: 30 bp)

		iAssembler	CAP3	MIRA	TGICL	Phrap	Newbler
No. unigenes		77,572	10,5103	127,565	80,540	70,489	69,301
Average unigene length (bp)		231.5	214.5	209.7	221	246.5	227.4
No. type I errors	identity < 97%	1	569	3	3,668	18,071	8,317
	overhang > 30 bp	1	11	2	1,621	5,066	11,266
No. type II errors		35	12,279	14,821	4,420	4,752	1,518
Total assembly errors		37	12,859	14,826	9,709	27,889	21,101
Run Time (minute)		227	79	57	101	43	7

We then tested performances of these assemblers using another set of parameters: minimum overlap length of 50 bp, minimum overlap percent identity of 95%, and maximum overhang length of 20 bp. The results also indicated that iAssembler generated much higher quality of assemblies than other assembly programs we investigated (Additional file 2).

We further evaluated the assemblers using a curated Arabidopsis EST dataset. Arabidopsis ESTs were downloaded from TAIR website ftp://ftp.arabidopsis.org/home/tair/Sequences/ATH_cDNA_EST_sequences_FASTA/ATH_EST_sequences_20101108.fas and aligned to Arabidopsis cDNAs (TAIR10; ftp://ftp.arabidopsis.org/home/tair/Sequences/blast_datasets/TAIR10_blastsets/TAIR10_cdna_20101214) using the megablast program with a minimum percent identity of 99 and a word size of 20. Only ESTs aligned to Arabidopsis cDNAs in their entire length were kept and the final collection contained 394,298 ESTs. These ESTs were assembled *de novo *using the six assemblers with the following parameters: minimum overlap length of 40 bp, minimum overlap percent identity of 97%, and maximum overhang length of 10 bp (Table 1). The resulting unigenes were then aligned back to Arabidopsis cDNAs and a small portion of the unigenes (~1%) could not be aligned to Arabidopsis cDNAs in their entire lengths (Table 4). Closer examination indicated that the majority of unaligned unigenes were those joined by two isoforms. This is not unexpected since in *de novo *EST assemblies it's inevitable that in certain cases two different isoforms could be joined together. iAssembler had slightly more unaligned unigenes than TGICL, MIRA, and CAP3, but much less than Phrap and Newbler. However, iAssembler corrected large number of type II errors found in other five assemblies, especially CAP3, MIRA and Phrap (Table 4). A significant number of type II errors found in CAP3, MIRA, TGICL, Phrap and Newbler assemblies were those with two nearly identical sequences with large overlaps (> 500 bp) failed to be assembled together, similar to the example shown in Additional File 1; while type II errors found in the iAssembler assembly were mainly due to sequence errors which caused two sequences not to be able to be aligned against each other (not meet the percent identity cutoff) but both of the sequences could be aligned to Arabidopsis cDNAs. In addition, iAssembler again generated significantly less type I assembly errors than TGICL, Phrap and Newbler, and approximately same as CAP3 and MIRA.

**Table 4:** Performances of assembly programs with a curated Arabidopsis EST dataset (minimum overlap: 40 bp, minimum overlap percent identity: 97%, maximum overhang: 10 bp)

	iAssembler	CAP3	MIRA	TGICL	Phrap	Newbler
No. unigenes	39,357	71,082	81,042	40567	70,364	41,930
Average unigene length (bp)	513.1	405.8	338.0	499.3	340.8	481.8
No. unigenes perfectly aligned to Arabidopsis cDNAs*	38,907	70,870	80,669	40,176	69,105	41,231
No. unigenes not perfectly aligned to Arabidopsis cDNAs	450	212	373	391	1,259	699
No. unigene pairs perfectly aligned to same Arabidopsis cDNAs with > = 40 bp overlaps (type II error)	465	28,630	41,696	1,729	34,735	4,587
No. ESTs and corresponding unigenes not aligned to same Arabidopsis cDNAs (type I error)	158	83	173	1,022	4,283	2,753

*perfectly aligned means that the sequences were aligned to Arabidopsis cDNAs in their entire lengths

In summary, our extensive evaluations of iAssembler and other EST assembly programs using different datasets and parameters support that iAssembler has significantly better performance, generating much less assembly errors in assembling Sanger and/or Roche-454 ESTs.

As shown in Table 2 and 3, the higher quality of assemblies achieved by iAssembler is a tradeoff of longer run time. The most time-consuming steps of iAssembler include the first initial assembly of EST sequences by MIRA and error detection by megablast. The run time of iAssembler can be significantly reduced by taking advantage of efficient usage of multi-threads by megablast and MIRA programs.

## Conclusion

In this study, we describe a standalone package called iAssembler, which can perform *de novo *assembly of ESTs generated by traditional Sanger and/or next-generation Roche-454 massively parallel pyrosequencing technologies. Through the use of an iterative assembly strategy and automated error detection and correction, iAssembler can deliver much higher accuracy in EST assembly than other existing EST assembly programs we investigated. Although iAssembler can only be executed under a command line interface, it's very easy to install and simple to use.

## Availability and requirement

Project name: iAssembler

Project home page: http://bioinfo.bti.cornell.edu/tool/iAssembler

Operating system(s): Linux

Programming language: Perl

Other requirements: Bioperl version 1.006 or higher

Third-party tools: BLAST, CAP3 and MIRA. These tools are already integrated into the iAssembler package.

License: None

Any restrictions to use by non-academics: none

## Authors' contributions

ZF conceived the general idea of this project and provided the guidance on the whole study. YZ developed the software and performed comparison with other assemblers. JG and LZ helped with design and evaluation of the software. ZF, YZ, and JG wrote the manuscript. All authors have read and approved the manuscript.

## Supplementary Material

Additional file 1 **Examples of common EST assembly errors**. The file provides several examples of common EST assembly errors. Click here for file

Additional file 2 **Performances of EST assembly programs**. The file provides evaluation results on performances of several EST assembly programs. Click here for file
