# Supplementary material for: iAssembler: a package for de novo assembly of Roche-454/Sanger transcriptome sequences
Source: BMC Bioinformatics. 2011 Nov 23;12:453. doi: 10.1186/1471-2105-12-453 (PMC3233632; doi:10.1186/1471-2105-12-453)
Supplement: Additional file 1 — Examples of common EST assembly errors. The file provides several examples of common EST assembly errors. [file 1471-2105-12-453-S1.DOC]

TC219875 1 -----------------------------------TGTGCCTAAACAAAATATGGCCACA
TC221582 1 ACGCTCGGTTTTTTGTCACACCTTTAGATATTCACTGTGCCTAAACAAAATATGGCCACA

TC219875 26 TCTCTCTCGGTTCGGCCCAACCGTACTATCATTAGCAGTGACCCGGCCGGTTCAACCCGG
TC221582 61 TCTCTCTCGGTTCGGCCCAACCGTACTATCATTAGCAGTGACCCGGCCGGTTCAACCCGG

TC219875 86 CCTAATCCAATCCGCCGCCCTCCAATCTCCGCTCATTTGCCGAAAATTCCAAACTCCGAT
TC221582 121 CCTAATCCAATCCGCCGCCCTCCAATCTCCGCTCATTTGCCGAAAATTCCAAACTCCGAT

TC219875 146 AGTTTCCGGCGGAGGAGGCTAGTGTTTACTTCTCACCGCCGGAAATTGAACCCCGCCGTT
TC221582 181 AGTTTCCGGCGGAGGAGGCTAGTGTTTACTTCTCACCGCCGGAAATTGAACCCCGCCGTT

TC219875 206 CAAGCAGGTTCTAGAGCTGATGATTCGGCTCCTTTCGAGATGTCGGTAGAGAATGCCCTG
TC221582 241 CAAGCAGGTTCTAGAGCTGATGATTCGGCTCCTTTCGAGATGTCGGTAGAGAATGCCCTG

TC219875 266 AAGTTGCTAGGTGTCACTGAGGGTGCTAGCTTCGATGATATTCTTCGTGCGAAAAAGTCA
TC221582 301 AAGTTGCTAGGTGTCACTGAGGGTGCTAGCTTCGATGATATTCTTCGTGCGAAAAAGTCA

TC219875 326 ATTATCTCTTCTTGTAAAGACGATCCAGATACAATTGCACAGGTAGAGGCGGCATATGAT
TC221582 361 ATTATCTCTTCTTGTAAAGACGATCCAGATACAATTGCACAGGTAGAGGCGGCATATGAT

TC219875 386 ATGCTTCTCATGCAAAGCTTATCACAGAGGAGATCTGGAAAAGTTGTGGATAGTAGTGTA
TC221582 421 ATGCTTCTCATGCAAAGCTTATCACAGAGGAGATCTGGAAAAGTTGTGGATAGTAGTGTA

TC219875 446 CGCTTTGCTGATGTTAAAGCTGCTAATGCTTCTGGGATGGGATCAATGCCCAAGTGGCTG
TC221582 481 CGCTTTGCTGATGTTAAAGCTGCTAATGCTTCTGGGATGGGATCAATGCCCAAGTGGCTG

TC219875 506 CAGACGACTGTCAAGAGCTCACCAGTCGCAGTTGAAGCACCAGCTTCTAAAGAGTTAGGT
TC221582 541 CAGACGACTGTCAAGAGCTCACCAGTCGCAGTTGAAGCACCAGCTTCTAAAGAGTTAGGT

TC219875 566 GTTCAAGCAGGAGTCTATGGAGCCTTAATGGTCTTAACATATGTTAATGGAGCCTCAACA
TC221582 601 GTTCAAGCAGGAGTCTATGGAGCCTTAATGGTCTTAACATATGTTAATGGAGCCTCAACA

TC219875 626 CCTTTAGGAGTATCGTATGGAGCTGATGTTCCTGGACTAATCTTAGCCACAAGCTTTGGG
TC221582 661 CCTTTAGGAGTATCGTATGGAGCTGATGTTCCTGGACTAATCTTAGCCACAAGCTTTGGG

TC219875 686 GCCACCTTGTACTTCATGACCAAGAAAAATGTCAAGTTGGGGAAAGCAAGCGTCATAACA
TC221582 ------------------------------------------------------------

**Figure S1.** Alignment of two tomato unigenes TC219875 and TC221582 according to the DFCI Tomato Gene Index.

TC237370 1 TGCTGCATGGTGGGTATAAGCGATCCGTGGAAATGGTACACATGCTGGCGCCAAACGCAA
AW218649 1 ------------------------------------------------------------

TC237370 61 TTCATGATGAGTGTCGTAGAAGACGTATGCAGGTAGCTGTAGTTGGTGTGCCAAAAACTA
AW218649 1 ------------GCCGTAAAAGACGTATAAAGGCAGCTGTAGTTGGTGTGCCAAAAACTA

TC237370 121 TAGACAATGATATTATGCTTATGGACAAAACATTTGGTTTTGATACTGCTGTTGAAGAAG
AW218649 49 TAGACAACGATATTATGCTTATGGATAAGACATTTGGTTTTGATACTGCCGTTGAAGAAG

TC237370 181 CACAGAGAGCCATTAATTCTGCTTATATTGAGGCACATAGTGCATATCATGGTATCGGAA
AW218649 109 CACAGAGAGCCATTAATTCTGCTTACATTGAGGCACACAGTGCATATCGTGGTATTGGAA

TC237370 241 TTGTGAAGTTGATGGGTCGTAGCAGTGGTTTTATAGCTATGCATGCATCACTAGCTAGTG
AW218649 169 TTGTGAAGTTGATGGGCCGTAGTAGTGGGTTTATAGCTATGCAAGCATCCCTAGCTAGTG

TC237370 301 GACAGATAGACATATGCTTGATTCCAGAGGTGCCTTTTAATCTGCACGGGCCTCATGGCG
AW218649 229 GACAAATAGACATATGCTTGATTCCAGAGGTGCCTTTCAATCTGCATGGCCCTCATGGTG

TC237370 361 TGTTGCAGCATCTGAAATATCTGCTTGAGACAAAGGGATCAGCTGTGATTTGTGTAGCAG
AW218649 289 TATTGAGGCATCTAAAATATCTGCTCTAGACAAAGGGATCAGCT----------------

TC237370 421 AGGGGGCAGGGCAGGATTTCCTTGAGAAAACCAATGCAAAGGATGCATCTGGAAATGCTG
AW218649 ------------------------------------------------------------

**Figure S2.** Alignment of a tomato EST (AW218649) against its corresponding unigene (TC237370) according to the DFCI Tomato Gene Index. Sequence identity between AW218649 and TC232370 is 91.5% and AW218649 is aligned to tomato chromosome 4 while TC237370 is aligned to tomato chromosome 11.

**A**


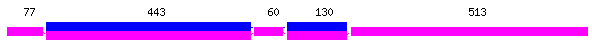


**AW031810**

**TC223103**

**B**

AW031810 ------------------------------------------------------------

TC223103 TGGAGGGCATTTTCTTCCACTTTCACTCTCACGCACACATTTCCTTAAATTGCCCTTCCC

AW031810 -----------------CTCTCTGAGAGAGAGTCTAAATTGGTCATCTCCACAATCAATG

TC223103 CTTTAACTGCCATCACCCTCTCTGAGAGAGAGTCTAAATTGGTCATCTCCACAATCAATG

AW031810 GCTGCCGCCGCCAGAATCTCCGCCTCCTCTACCTCACGAACTTTTTATTTCCGTCATTCA

TC223103 GCTGCCGCCGCCAGAATCTCCGCCTCCTCTACCTCACGAACTTTTTATTTCCGTCATTCA

AW031810 CCGTTTCTTGGCCCAAAACCTACTTCGACAACCTCACATGTTTCTCCAATCTCTCCTTTT

TC223103 CCGTTTCTTGGCCCAAAACCTACTTCGACAACCTCACATGTTTCTCCAATCTCTCCTTTT

AW031810 TCTCTTAATCTAGGCCCAATTTTGAGGTCTAGAAGAAAACCCAGTTTCACTGTTTGCTTT

TC223103 TCTCTTAATCTAGGCCCAATTTTGAGGTCTAGAAGAAAACCCAGTTTCACTGTTTGCTTT

AW031810 GTTCTCGAGGATGAGAAGCTGAAACCTCAATTTGACGATGAGGCTGAGGATTTTGAAAAG

TC223103 GTTCTCGAGGATGAGAAGCTGAAACCTCAATTTGACGATGAGGCTGAGGATTTTGAAAAG

AW031810 AAGATTGAGGAACAGATCTTAGCTACTCGCTTGGCGGAGAAACTGGCTAGGAAGAAATCG

TC223103 AAGATTGAGGAACAGATCTTAGCTACTCGCTTGGCGGAGAAACTGGCTAGGAAGAAATCG

AW031810 GAGAGGTTCACTTATCTTGCGGCTGCTATAATGTCTAGTTTGGGGATTACTTCTATGGCT

TC223103 GAGAGGTTTACTTATCTTGTGGCTGCTATAATGTCTAGTTTTGGGATTACTTCTATGGCT

AW031810 GTTATGGCTGTTTATCACAGATTTTCGTGGCAAATGGAGG--------------------

TC223103 GTTATGGCTGTTTATTACAGATTTTCGTGGCAAATGGAGGGAGGAGAAGTTCCTGTAACC

AW031810 ----------------------------------------TAGAAATGGA-TTTTGGGCG

TC223103 GAAATGTTGGGTACATTTGCTCTCTCTGTTGGTGCTGCTGTAGGAATGGAGTTTTGGGCG

AW031810 AGATGGGCACACAAAGCACTGTGGCATGCTTCACTATGGCACATGCATGAGTCACACCAC

TC223103 AGATGGGCACACAAAGCACTGTGGCATGCTTCACTATGGCACATGCATGAGTCACACCAC

AW031810 AAACCAAGAGAAGGACCTTTTGAGCTGAACGACGTTTTCGCCATAACAAA----------

TC223103 AAACCAAGAGAAGGACCTTTTGAGCTGAACGACGTTTTCGCCATAACAAACGCTGTTCCA

AW031810 ------------------------------------------------------------

TC223103 GCAATAGCCCTCCTCAACTATGGTTTCTTCCATAAAGGCCTCATTGCCGGACTATGCTTC

**Figure S3.** Alignment of a tomato EST (AW031810) against its corresponding unigene TC223103 according to the DFCI Tomato Gene Index. **(A)** Schematic representation of the alignment. Numbers indicate the length (bp) of the corresponding aligned blocks. **(B)** Detailed sequence alignment of AW031810 and TC223103
