# Supplementary material for: iAssembler: a package for de novo assembly of Roche-454/Sanger transcriptome sequences
Source: BMC Bioinformatics. 2011 Nov 23;12:453. doi: 10.1186/1471-2105-12-453 (PMC3233632; doi:10.1186/1471-2105-12-453)
Supplement: Additional file 2 — Performances of EST assembly programs. The file provides evaluation results on performances of several EST assembly programs. [file 1471-2105-12-453-S2.DOC]

**Table S1. Performances of assembly programs with olive Roche-454 ESTs (minimum overlap: 50 bp, minimum overlap percent identity: 95%, maximum overhang: 20 bp)**

| **Type** | | **iAssembler** | **CAP3** | **MIRA** | **TGICL** | **Phrap** | **Newbler** |
| --- | --- | --- | --- | --- | --- | --- | --- |
| No. unigenes | | 75,489 | 102,970 | 115,870 | 80282 | 70,489 | 69,140 |
| Average unigene length (bp) | | 229.1 | 211.8 | 208.8 | 220 | 246.5 | 226.8 |
| No. type I errors | identity < 95% | 0 | 39 | 1 | 539 | 8,318 | 4,156 |
|  | overhang > 20 bp | 5 | 120 | 12 | 3,003 | 8,166 | 13,794 |
| No. type II errors | | 40 | 11,838 | 12,588 | 4,660 | 4,527 | 784 |
| Total assembly errors | | 45 | 11997 | 12601 | 8,202 | 21,011 | 18,734 |
| Run Time (minute) | | 194 | 79 | 49 | 103 | 43 | 7 |

**Table S2. Performances of assembly programs with tomato Sanger ESTs (minimum overlap: 50 bp, minimum overlap percent identity: 95%, maximum overhang: 20 bp)**

|  | | **iAssembler** | **CAP3** | **MIRA** | **TGICL** | **Phrap** | **Newbler** |
| --- | --- | --- | --- | --- | --- | --- | --- |
| No. unigenes | | 51,745 | 83,689 | 74,465 | 50,885 | 43,434 | 48,981 |
| Average unigene length (bp) | | 945 | 765.1 | 785.2 | 927.5 | 963.7 | 985.7 |
| No. type I errors | identity < 95% | 0 | 20 | 13,499 | 292 | 5,492 | 3,177 |
|  | overhang > 20 bp | 6 | 983 | 13,657 | 9,147 | 41,472 | 28,346 |
| No. type II errors | | 337 | 13,564 | 9,827 | 3,894 | 4,019 | 5,937 |
| Total assembly errors | | 343 | 14,567 | 36,983 | 13,333 | 50,983 | 37,460 |
| Run Time (minute) | | 621 | 365 | 251 | 465 | 175 | 42 |
